# Characterization of the anti-inflammatory *Lactobacillus reuteri* BM36301 and its probiotic benefits on aged mice

**DOI:** 10.1186/s12866-016-0686-7

**Published:** 2016-04-19

**Authors:** Joon Lee, Woo Yang, Andrew Hostetler, Nathan Schultz, Mark A. Suckow, Kay L. Stewart, Daniel D. Kim, Hyung Soo Kim

**Affiliations:** Research and Development, Benebios LLC, 10527 Garden Grove Blvd, Garden Grove, CA 92843 USA; 400 Freimann Life Science Center, University of Notre Dame, Notre Dame, IN 46556 USA; Current address: Department of Veterinary Population Medicine, 225 Veterinary Medical Center, University of Minnesota, 1365 Gortner Ave, St. Paul, MN 55108 USA

**Keywords:** Probiotics, *L. reuteri*, Lactic acid bacteria, Anti-inflammatory, TNF-α, C57BL/6 mice, Aging, Testosterone, Skin health, Hair growth

## Abstract

**Background:**

The gut microbiota is playing more important roles in host immune regulation than was initially expected. Since many benefits of microbes are highly strain-specific and their mechanistic details remain largely elusive, further identification of new probiotic bacteria with immunoregulatory potentials is of great interest.

**Results:**

We have screened our collection of probiotic lactic acid bacteria (LAB) for their efficacy in modulating host immune response. Some LAB are characterized by suppression of TNF-α induction when LAB culture supernatants are added to THP-1 cells, demonstrating the LAB’s anti-inflammatory potential. These suppressive materials were not inactivated by heat or trypsin. On the other hand, treatment of THP-1 directly with live bacterial cells identified a group of pro-inflammatory LAB, which stimulated significant production of TNF-α. Among those, we chose the *Lactobacillus reuteri* BM36301 as an anti-inflammatory strain and the *L. reuteri* BM36304 as a pro-inflammatory strain, and further studied their in vivo effects. We supplied C57BL/6 mice with these bacteria in drinking water while feeding them a standard diet for 20 weeks. Interestingly, these *L. reuteri* strains evoked different consequences depending on the gender of the mice. That is, males treated with anti-inflammatory BM36301 experienced less weight gain and higher testosterone level; females treated with BM36301 maintained lower serum TNF-α as well as healthy skin with active folliculogenesis and hair growth. Furthermore, while males treated with pro-inflammatory BM36304 developed higher serum levels of TNF-α and insulin, in contrast females did not experience such effects from this bacteria strain.

**Conclusion:**

The *L. reuteri* BM36301 was selected as an anti-inflammatory strain in vitro. It helped mice maintain healthy conditions as they aged. These findings propose the *L. reuteri* BM36301 as a potential probiotic strain to improve various aspects of aging issues.

**Electronic supplementary material:**

The online version of this article (doi:10.1186/s12866-016-0686-7) contains supplementary material, which is available to authorized users.

## Background

The collections of commensal microbes, called microbiota, are superior to their host in number as well as in genetic and metabolic diversity [1]. Particularly the gut microflora, which comprises more than 90 % of all commensals, have profound effects on their host in the development and maintenance of body systems, including metabolism, immune regulation, or even neuronal function [1, 2]. There are an increasing number of reports suggesting that a disruption in the normal composition of microbial communities can cause a wide array of health issues [3–7]. These include obesity, diabetes, metabolic syndrome, inflammatory bowel disease (IBD), autoimmune disease, colon cancer, or even depression and neurodevelopmental disorders [3, 4, 8–10]. Intriguingly, oral supplementation of selective bacteria to individuals suffering from health problems has shown to be effective in ameliorating the symptoms or curing the disease, in part by rebalancing the gut microbial composition [8, 9, 11, 12]. As a result, these beneficial microbes (or probiotics) and their metabolites have come under great interest with respect to their therapeutic potential. Accordingly, lactic acid bacteria (LAB), which have been widely used in food processing for a century, are now under re-evaluation in regard to their probiotic efficacy [6, 11, 13].

In order for dietary probiotics to effectively function in the gut, they should meet certain basic qualifications [14–17]. First, they must be able to withstand the challenging environment of gastric acids, digestive enzymes, or bile salts in the stomach and intestines. Also, their intrinsic antimicrobial activities against pathogens would be beneficial to hosts. Finally, it is desirable that they can adhere well enough to the mucosal layer in the gastrointestinal (GI) tract, especially since they need to compete with other pathogens for nutrient uptake.

Probiotics contain not only these general health advantages but also other far more diverse benefits. In particular, the involvement of gut microbes in the regulation of host immune function is extraordinarily comprehensive [1, 11, 13, 18–20]. Many studies have used germ-free or antibiotic-treated mice to compare the susceptibility of developing immune disease with that of conventionally grown or non-treated mice, respectively. The current understanding of the roles of gut microbes in host immune regulation can be briefly summarized as follows. First, the gut flora contributes to the development of various lymphoid cells, including T_H_17 cells, T_Reg_ Cells, IgA-producing plasma cells, and innate lymphoid cells. Second, it confers resistance against pathogenic bacteria or viruses by enhancing innate and adaptive immunities. Third, the gut flora influences development of intestinal disease. For example, IBD can be stimulated by unprecedented overgrowth of pathogenic symbionts. In this case, the homeostasis between beneficial bacteria and pathogens are critical to minimize the inflammatory shift. Finally, the gut flora has the capacity to affect even extra-intestinal diseases, such as multiple sclerosis, arthritis, type 1 diabetes, allergic inflammation, and cancers [18, 19]. These systemic effects even extend to affect skin health, skin wound healing, eczema, and endocrine pathways predominantly via immune networks [21, 22].

Local interactions between the gut microbiota and its host primarily involve the direct recognition of bacterial surface molecules by neighboring epithelial cells and immune cells in the intestine [13, 18]. For example, lipopolysaccharide (LPS), peptidoglycan, lipoteichoic acids, and exopolysaccharides of certain bacterial cell walls can induce a variety of gut immune responses. Expression and modification of these compartments depend on bacterial genes and metabolic activities, which explains the strain-specificity. Meanwhile, remote interactions can be initiated by secretion of various bacterial metabolic products [13]. For example, organic acids such as L-lactic acid (from *Lactobacillus casei* strain Shirota) and short-chain fatty acids (SCFAs) (from various gut bacteria), polyamines (from *Bifidobacterium animalis* subsp. *lactis* strain LKM512), small metabolites such as AI-3 (from commensal *E. coli*) and histamine (from *L. reuteri* strain ATCC PTA 6475), or even proteins such as p40/p75 (from *Lactobacillus rhamnosus* strain GG) were reported for their remote immunoregulatory roles [23–27].

As an effort to search for immunomodulatory microbes, in vitro tissue cultures using myeloid cells such as THP-1, peripheral blood mononuclear cells (PBMCs), dendritic cells (DCs), or intestinal epithelial cells (IECs) have been widely employed [28]. Common cytokines studied include tumor necrosis factor α (TNF-α), interleukins 8, 12, and 17 (IL-8, IL-12, and IL-17), all of which are known to induce many inflammatory responses, primarily pro-inflammatory. In contrast, transforming growth factor β (TGF-β) and IL-10 are frequently assessed as anti-inflammatory markers. For example, culture supernatants from various LAB were shown to contain metabolites that suppress the expression of TNF-α or enhance the expression of IL-10 in various in vitro tissue culture systems [29, 30]. Also, direct treatment of immune cells with live bacteria resulted in the secretion of various cytokines [31]. These studies have established in vitro approaches to identify immunoregulatory microbes and metabolites [13].

Since these regulatory functions of LAB are highly strain-specific, great potential for further characterization of LAB remains. In this study, we established a protocol to isolate probiotic LAB with potential immunoregulatory ability. We compared both anti-inflammatory and pro-inflammatory activities of each bacterial strain in vitro. Finally, we applied the elected LAB to a mouse model system to verify their relevance with respect to the in vitro screening.

## Results

### Characterization of probiotic lactic acid bacteria

We have been isolating lactic acid bacteria (LAB) for years from various sources including humans, animals, plants, and food products. These *B*enebios *M*icroorganisms (BM) collections are comprised of over 500 strains, with probiotic potentials revealed from their initial screenings. In this study, we chose four human commensal strains derived from fecal samples and further studied them in detail (Table 1). Those specifically examined were two *Lactobacillus reuteri* strains (BM36301 and BM36304), a *L. gasseri* strain (BM33601), and a *Bifidobacterium animalis* subsp. *lactis* strain (BM10307).

**Table 1:** Characterization of Probiotic Lactic Acid Bacteria

Strain	Taxonomy	Resistance against ^a^		Antimicrobial activity^b^	Adhesion to IEC^c^
		Acids (pH 2.5)	Bile salts (0.3 %)		
BM36301	*L. reuteri*	50.2 %	53.4 %	+	5.5 ± 0.4
BM36304	*L. reuteri*	83.5 %	89.4 %	++	7.4 ± 0.8
BM33601	*L. gasseri*	98.5 %	72.6 %	±	1.7 ± 0.2
BM10307	*B. animalis* subsp. *lactis*	43.0 %	47.3 %	+	0.3 ± 0.05

^a^The survival rate was calculated by comparing the colony forming unit (CFU) of treated culture with that of untreated control. A representative data from more than three independent experiments is shown

^b^Clear zonal inhibition of the growth of the tester strain was measured from the edge of the spot (see Methods) and expressed as; ± (less than 0.5 mm inhibitory zone), + (0.5 ~ 1.0 mm), or ++ (1.0 ~ 3.0 mm)

^c^Average bacterial count per HT-29 cells is listed with standard deviation (± SD)

We examined the bacteria with respect to their general qualifications as probiotics (Table 1). First, these BM strains showed resistance against acids (pH 2.5) and bile salts (0.3 %), demonstrating a 43 % to 98.5 % survival rate range after 1 h treatment at 37 °C, which are reasonably high scores [14]. Next, we tested the antimicrobial activities against enteric bacteria. While *L. gasseri* BM33601 showed a minimal inhibition of the tester strain *E. coli*, all others formed distinct inhibitory zones (0.5–3 mm distance from LAB edges). These inhibitory effects are usually caused by bacteriocins, organic acids (lactic acid or acetic acid), hydrogen peroxide, or ethanol from the LAB [32, 33]. Finally, we looked at their ability to bind to human intestinal epithelial cells (IECs) in vitro. For this purpose, we cultivated human colon cancer cells, HT-29, on coverslips for 15 days and applied live LAB cultures onto them for 1 h (Methods). After extensive washing, the remaining bacterial cells were visualized by Gram staining for counting under the microscope [34]. The *L. reuteri* strains BM36301 and BM36304 showed high retention scores (5.5–7.4 bacteria per HT-29), while *L. gasseri* BM33601 had moderate (1.7 bacteria per HT-29) and *B. animalis* subsp. *lactis* BM10307 had low (0.3 bacteria per HT-29) scores. Stronger adhesion of LAB to IECs is particularly important since they need to stay at the gut mucosal layer long enough for the benefits to take effect on the host [16, 17, 34]. From these primary experiments, the two *L. reuteri* strains revealed higher potential as quality probiotics.

### Regulation of TNF-α production by lactic acid bacteria in vitro

**Fig. 1:**
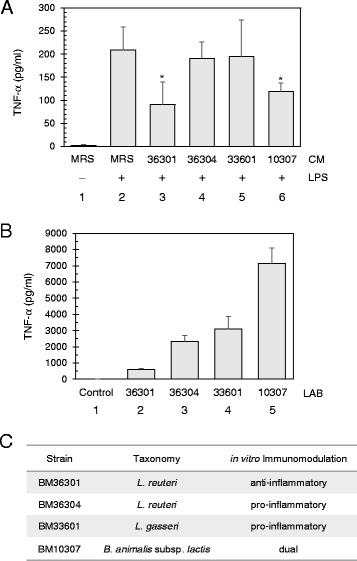
Immunomodulation by lactic acid bacteria in vitro. **a** Screening of lactic acid bacteria (LAB) with conditioned medium (CM) for anti-inflammatory activities against LPS-induced TNF-α production in THP-1. Without LPS, a non-detectable amount of TNF-α was observed (lane 1). With 150 ng/ml of LPS treatment, THP-1 cells produced significant amounts of TNF-α (lane 2). Each CM was treated to 5 % of the THP-1 culture volume to assess its inhibitory effects (lane 3, *L. reuteri* BM36301; lane 4, *L. reuteri* BM36304; lane 5, *L. gasseri* BM33601; and lane 6, *B. animalis* subsp. *lactis* BM10307). CM from control medium (MRS) was prepared and added in lanes 1 and 2. * indicates p < 0.03 from the t-test between the control (lane 2) and either BM36301 CM-treated (lane 3) or BM10307 CM-treated (lane 6). Bars show averages with standard deviation (SD) from 5 independent assays. **b** Screening of LAB with live cells for pro-inflammatory activities to induce TNF-α production in THP-1 cells. About 1.5 × 10^8^ bacterial cells from exponentially growing cultures were applied to 6 × 10^5^ THP-1 cells for 6 h. The cell-free supernatants were collected and assessed for the secreted TNF-α by ELISA method. The results show averages with SD from 4 independent experiments. **c** Summary of in vitro immunomodulation by various lactic acid bacteria

We aimed to screen LAB for their potential to suppress intestinal inflammation. To this end, we adapted an in vitro tissue culture system where the human myeloid cells (THP-1) secrete TNF-α upon activation of their Toll-like receptors (TLR) by bacterial lipopolysaccharide (LPS) [28, 29]. First, we verified that THP-1 cells of 5 × 10^4^ in 1 ml culture treated with LPS at 150 ng/ml dosage for 3.5 h usually resulted in a production of 200–300 pg/ml TNF-α (Fig. 1a, lanes 1 and 2). Next, we collected the supernatants of bacterial cultures grown for 24 h, vacuum-dried them, and reconstituted the *c*onditioned *m*edium (CM) (Methods). This CM contains complex activities to both suppress and induce TNF-α, depending on the LAB and culture conditions [23, 29]. Indeed, we observed slight production of TNF-α by the CMs from BM36304 and BM36301 without LPS, but this amount was less than 20 % of LPS-stimulated induction (data not shown). Finally, we added each CM up to 5 % of THP-1 culture (v/v) in the presence of LPS (Fig. 1a, lanes 3–6). The TNF-α production with LPS (208 ± 49 pg/ml, lane 2) was suppressed to 40 % with the CM from *L. reuteri* BM36301 (90.90 ± 49.3 pg/ml, lane 3), and to 52 % with the CM from *B. animalis* subsp. *lactis* BM10307 (118.8 ± 19.0 pg/ml, lane 6). For our screening purposes, we considered the suppression of TNF-α close to or less than 50 % of expression by LPS treatment as anti-inflammatory. However, the CMs from *L. reuteri* BM36304 and *L. gasseri* BM33601 were not able to suppress the TNF-α production to that point. We verified this inert activity by preparing these CMs under various culture conditions (data not shown).

Next, we examined the TNF-α induction capacity of the bacterial cells themselves because cellular components of the Gram positive bacteria may induce inflammatory responses [13, 31]. For this purpose, we grew bacteria to an exponential phase (16 h), then harvested, washed, and added these live bacterial cells in excess (250-fold cell counts) to the THP-1 culture. Metabolic activities of bacterial cells were kept minimized by antibiotics added in the THP-1 medium. After 6 h of incubation, the secreted TNF-α was quantitatively measured (Fig. 1b). Even though all four LAB cells were found to induce TNF-α, BM36301 produced significantly smaller amount (609 pg/ml) than the others: BM36304 (2342 pg/ml), BM33601 (3100 pg/ml), and BM10307 (7141 pg/ml). During this 6-hr co-incubation, we observed that THP-1 cells experienced variable cell death depending on the LAB used (Additional file 1). Most notably, BM36301, the lowest TNF-α inducer, caused the least amount of THP-1 death (9.7 %), while the other higher TNF-α inducing strains caused significantly more prominent death (24–38 %). Therefore, it is not the case that BM36301 induced low TNF-α due to the loss of THP-1 viability per se. For our screening purposes, we considered LAB with active TNF-α production of more than 1000 pg/ml as pro-inflammatory.

In summary, the in vitro immunomodulation activities were assigned as anti-inflammatory with the suppressive CM (BM36301 and BM10307) and pro-inflammatory with the inductive live cells (BM36304, BM33601, and BM10307). Since BM10307 showed both activities, we assigned it as possessing dual functionality (Fig. 1c). It was interesting to note that the two *L. reuteri* strains displayed distinct activities. From the initial screening of our wide range of BM collections, the anti-inflammatory strains proved to be rather rare, with a discovery rate of less than 5 % (data not shown). Also some strains showed neither activity, thus falling into the category of ‘neutral’ (data not shown).

### Suppressive molecules in the CMs are stable against heat and trypsin

**Fig. 2:**
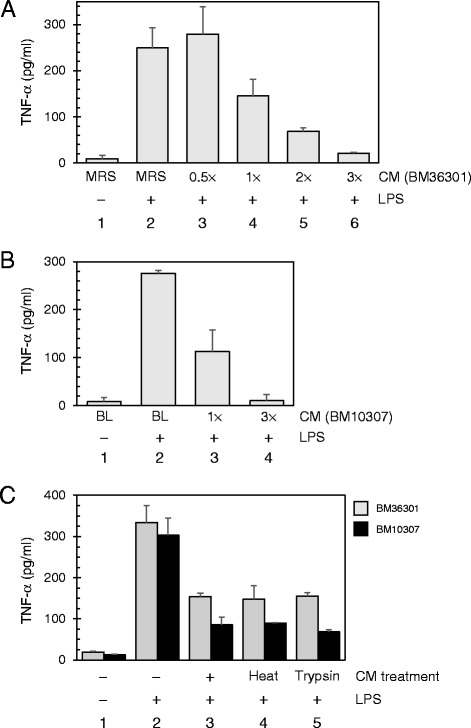
Suppressive CMs are quantitative and resistant against heat and enzymatic digestion. **a** CM from *L. reuteri* strain BM36301 represses TNF-α production from LPS-treated THP-1 cells in a quantitative manner. TNF-α levels produced from the THP-1 cultures with (lanes 2 – 6) or without (lane 1) LPS were measured by ELISA method. For treatment of CM, 2.5 % (0.5×, lane 3), 5 % (1×, lane 4), 10 % (2×, lane 5), or 15 % (3×, lane 6) of THP-1 culture volume (v/v) was added at the time of LPS stimulation. CM from the control medium (MRS) was added to 5 % in lanes 1 and 2. The data show averages with SD from 3 independent assays. **b** CM from *B. animalis* subsp. *lactis* strain BM10307 contains metabolites that repress TNF-α production from LPS-treated THP-1 cells in a quantitative manner. The LPS and CM were treated as indicated. CM from the control medium (BL) was added to 5 % in lanes 1 and 2. Data are from 3 independent experiments. **c** CMs from BM36301 (gray) or BM10307 (black) were either boiled (lane 4) or treated with trypsin (lane 5) to compare their activities with native CMs (lane 3). Results are averages with SD from 3 independent experiments

Since the CM from LAB is composed of various bacterial metabolites as well as cellular components, we sought to better understand the anti-inflammatory nature of the CM. The expression of TNF-α quantitatively diminished with increasing concentrations of the CMs from *L. reuteri* BM36301 (Fig. 2a) and *B. animalis* subsp. *lactis* BM10307 (Fig. 2b). Interestingly, a smaller amount of CM from BM36301 (0.5× input or 2.5 % v/v) actually induced TNF-α further, consistent with the observation that this CM contains TNF-α-inducing materials to some extent (Fig. 2a, lanes 2 and 3). Nonetheless, the suppressive effects became dominant in the face of higher amounts of CM, suggesting that the anti-inflammatory materials are quantitatively additive and that the corresponding receptors on the THP-1 cell surface are abundant enough to readily respond to increasing CM treatment. Overall, these observations suggest that the CMs contain active metabolites responsible for TNF-α suppression, in spite of their complex nature.

We further examined the physicochemical characteristics of anti-inflammatory materials in the CMs. Boiling the CMs from each strain for 10 min was found to be ineffective in altering their TNF-α inhibitory functions compared with native CMs (Fig. 2c, lanes 3 and 4). Likewise, trypsin treatment did not affect their activities (Fig. 2c, lane 5). These observations suggest that the main inhibitory factors in these CMs are neither proteins nor heat-sensitive molecules.

### Regulation of TNF-α in mice with probiotic bacteria

The ultimate question in regard to in vitro TNF-α regulation based on our LAB screening was how relevant it would be with the in vivo applications. We directly sought to answer this question by applying the selected *L. reuteri* strains to a mouse model system. We prepared 20-week-old, inbred C57BL/6 mice of 6 males and 8 females per group. Then over a 20-week period, one group was treated with the anti-inflammatory BM36301, another group was treated with the pro-inflammatory BM36304, and the control group was treated with dextrose (Methods). As a means to minimize distress on the animals, the LAB cultures were administered via drinking water to meet the daily consumption dosage of 1 × 10^6^ bacteria per mouse. We also provided a standard diet so that the aging process was natural during the period. At the end of the 20-week incubation (total 40 weeks old age), we euthanized the mice and withdrew blood for serum preparation to measure cytokines and testosterone using quantitative ELISA methods. Upon performing necropsy, we found no gross morphologic abnormalities.

**Fig. 3:**
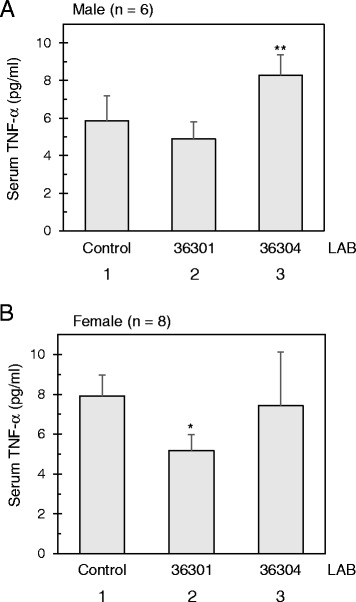
TNF-α from mice C57BL/6 supplemented with probiotic bacteria. **a** The TNF-α of the serum from male mice (n = 6 each) each fed with *L. reuteri* strain. ** indicates *p* = 0.006 from the t-test in comparison with the control group (lanes 1 and 3). **b** The TNF-α of the serum from female mice (n = 8 each) each fed with *L. reuteri* strain. * indicates *p* = 0.017 from the t-test in comparison with the control group (lanes 1 and 2)

We were primarily interested in the serum TNF-α levels from these mice. As shown in Fig. 3a, control males showed 5.85 ± 1.34 pg/ml of serum TNF-α. BM36301-treated males showed a slight reduction (4.90 ± 0.9 pg/ml), though without meaningful significance (*p* = 0.59). However, BM36304-treated males maintained significantly higher TNF-α levels (8.28 ± 1.09 pg/ml, *p* = 0.006 vs. control). We also quantitated the female’s TNF-α concentrations (Fig. 3b). We found that the female control group retained higher TNF-α levels (7.9 ± 1.05 pg/ml) than the male control group, though without significance (*p* = 0.42). Most notably, BM36301-fed females displayed significantly reduced amounts of TNF-α (5.17 ± 0.80 pg/ml, *p* = 0.017 vs. control), while BM36304-treated females showed similar levels with the control mice (7.43 ± 2.69 pg/ml, *p* = 0.71 vs. control). Unlike these variations of TNF-α, we could not discern meaningful differences of serum IL-10 levels between the groups (data not shown).

In summary, we observed a reduction of serum TNF-α with the anti-inflammatory BM36301, with a more pronounced effect observed in females. The pro-inflammatory BM36304 led to an increase of TNF-α in males, but not in females. These findings suggest that our in vitro screening carries meaningful relevance with in vivo maintenance of TNF-α in these mouse trials.

### *L. reuteri* BM36301 maintained reduced body weight gain and high testosterone in male mice

**Fig. 4:**
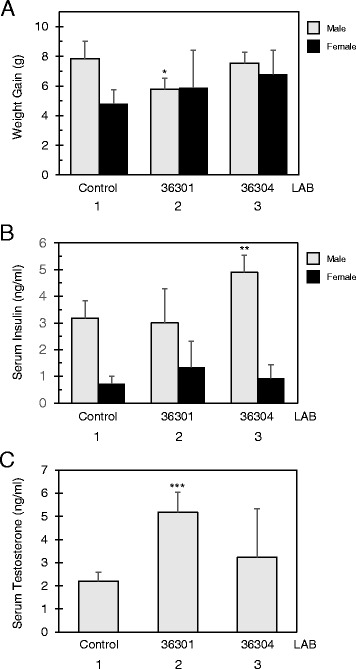
Aged male mice supplemented with *L. reuteri* strain BM36301 maintain healthy body index. **a** Net weight gain of mice C57BL/6 (n = 6 for male, grey bar; n = 8 for female, dark bar) fed with each *L. reuteri* strain for 20 week period. * indicates *p* = 0.040 from the t-test in comparison with the control group (male lanes 1 and 2). **b** Serum insulin concentration from mice fed with each *L. reuteri* strain as in (**a**). ** indicates *p* = 0.027 from the t-test in comparison with the control group (male lanes 1 and 3). **c** Serum testosterone amount from male mice fed with each *L. reuteri* strain as in (**a**). *** indicates *p* = 0.0025 from the t-test in comparison with the control group (lanes 1 and 2)

Aging is the source of many health issues in humans, including diabetes and obesity. In order to exacerbate such aging issues in the animal model, high fat diets are often employed [35, 36]. However, the interpretation on those accelerated experiments can be complicated since no aging in humans should be driven by a purposed high fat diet. We cultivated all the mice with a standard diet so that their aging process could be most natural. In the 20-week experiment timespan starting from the age of 20 weeks old, control group males gained about 8 g weight (7.83 ± 1.2 g) and control group females gained about 5 g weight (4.8 ± 0.94 g). Remarkably, it was noticeable that the weight gain in males fed with the anti-inflammatory BM36301 was significantly lower than in the control (5.78 ± 0.75 g, *p* = 0.040) by 36 % (Fig. 4a, lanes 1 and 2). However, the weight gain of BM36304-treated males (7.53 ± 0.74 g) was not significantly different from the control (*p* = 0.67). The weight gains of the female groups were not statistically different from each other.

Another significant difference was the serum insulin level from BM36304-treated males (Fig. 4b). While the control male group showed 3.18 ± 0.65 ng/ml of insulin, the BM36304-fed male group showed 4.91 ± 0.63 ng/ml of insulin (*p* = 0.027). However, consistent with the finding that the weight gain from this group was not much different from the control (Fig. 4a), we did not observe a more prominent abdominal fat accumulation in BM36304-treated males (data not shown). At 40 weeks old, the insulin spike may not have caused clinically-evident diabetes in the mice, but the disease may have become more pronounced if the mice aged further. The insulin variation between the female groups was not significantly different from each other.

Finally, we focused on the males’ testosterone levels. As shown in Fig. 4c, the anti-inflammatory BM36301-fed males retained significantly higher levels of serum testosterone (5.18 ± 0.87 ng/ml) than the control group (2.20 ± 0.38 ng/ml, *p* = 0.0025). However, the BM36304-treated group was not statistically different (3.23 ± 2.10 ng/ml, *p* = 0.07) from the control. In order to further understand the testosterone results, we examined the testicles of each mouse. The weight of paired testes of the BM36301-fed males was 0.579 ± 0.075 g, 10 % heavier than that of the control’s (0.525 ± 0.05 g, *p* = 0.049). Considering the smaller weight gain in the BM36301 group (Fig. 4a), this size difference of the testes is more evident; the ratio of testes-weight to body-weight-gain (TW/WG) of the control was only 67 % of that of the BM36301 group. However, we could not observe microscopic differences of the tissues from the testes, such as the diameter of seminiferous tubules between these two groups (156.03 ± 6.65 μm of control vs. 153.95 ± 18.66 μm of BM36301-treated males, *p* = 0.51). We have not performed other studies such as comparing spermatogenesis or Leydig cell area.

In summary, the anti-inflammatory BM36301 helped males maintain lower weight gain with bigger testes and more testosterone as they aged. Reduced testosterone production in aged males has been proposed to be related with age-dependent lesions in the testes, presumably as a result of inflammatory insult [36, 37]. Meanwhile, the pro-inflammatory BM36304 triggered an increase of insulin, though without changes in weight gain or testosterone among males. Notably, none of these bacterial strains had meaningful influences on these body measures in female mice.

### Healthy female skin from anti-inflammatory BM36301

**Fig. 5:**
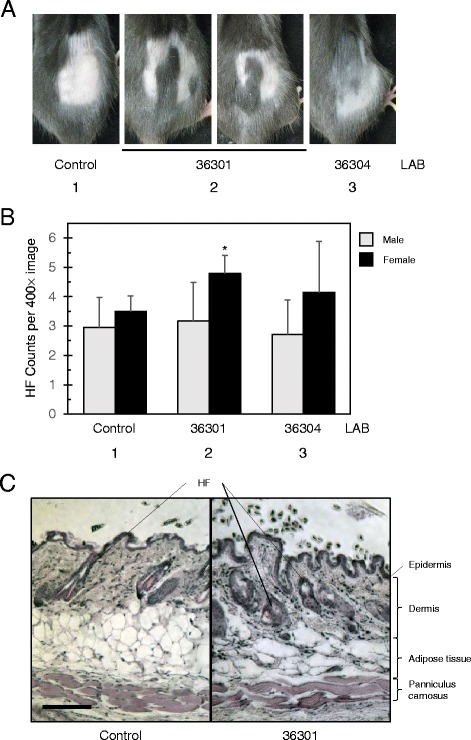
Aged female mice supplemented with *L. reuteri* strain BM36301 display healthy skins. **a** Hair re-growth experiment on C57BL/6 mice consuming *L. reuteri* strain-treated water or control water. A 2 × 2 cm wide area of skin was clearly shaved and the hair re-growth was examined a week later. **b** Hair follicle (HF) counts were collected from the skin section samples of mice fed with either control or *L. reuteri* strains. Five microscopic images at 400× resolution from 5 to 8 mice were counted. * indicates *p* = 0.023 from the t-test in comparison with the control group (female lanes 1 and 2). **c** Skin sections from control (left) and BM36301-treated (right) mice were stained with Hematoxylin and Eosin to show skin tissue layers and HFs as labeled. Photos were taken at 100× resolution; the bar indicates of 100 μm in length for scale

One interesting feature of certain probiotics is that they can promote skin health in a manner dependent on immune regulation [21, 38]. Since the BM36301 also showed anti-inflammatory effects in vitro and in vivo, we asked the question whether this strain can provoke such health benefits in skin. At the 18^th^ week of treatment, we shaved an area on 2 mice from each group and examined them after 1 week. Interestingly, BM36301-fed female mice exhibited faster hair re-growth, though not full recovery, at the shaved area (Fig. 5a). In contrast, control or BM36304 groups did not display such a rapid pace of recovery. None of the treated males showed hair re-growth distinguishably faster than the control, indicating that this accelerated hair growth may be obvious in only females. Another metric to evaluate skin health is by examining fur shininess. However, we did not observe meaningful differences between each group through sensory or light meter measurements, mainly due to the relatively shiny fur conditions of the controls (data not shown).

We further assessed skin health by examining skin cross-sections under the microscope after staining with Hematoxylin and Eosin. As shown in Fig. 5b and c, we found that the BM36301-treated female mice showed higher counts of subcutaneous hair follicle (HF) than control female mice. The HF counts per microscopic view at 400× resolution were 3.50 ± 0.52 for the control female group, while the counts were 4.80 ± 0.61 from BM36301-fed females with a meaningful difference (*p* = 0.023). However, BM36304-treated females presented only a slight change (4.15 ± 1.73, *p* = 0.55 vs. control). Upon a more detailed analysis of the hair follicles, we found that the BM36301-fed females displayed marginally more active hair cycle stages than the control (anagen + catagen: telogen was 81:19 from control vs. 95: 5 from BM36301-fed females). Meanwhile, the HF counts from the male groups were not much different from each other (Fig. 5b). Finally, we also assessed the skin thickness measured as the depth from the epidermis to panniculus carnosus (Fig. 5c, Additional file 1). We could not find much difference of the depth between the control and the BM36301-treated females. However, the dermal layers of BM36301-fed mice proved marginally deeper than the controls’, and the adipose tissue from BM36301-treated mice was shallower than that that of the control. Most of the subcutaneous HFs were found in the dermis.

Overall, these observations suggest that the treatment with anti-inflammatory BM36301 fostered healthier skin as evidenced by hair re-growth and HF counts in females. However, the microscopic differences of skin samples between the control and the BM36301 groups appeared rather marginal. These skin health benefits were not observed in males. Also, the pro-inflammatory BM36304 did not cause any adverse effects on the skin at the time of experiments.

## Discussion

In this study, we have examined the health benefits of the *L. reuteri* BM36301 strain. This strain met selection criteria for further in vitro and in vivo studies (Table 1). From an anaerobic cultivation, this strain produced a culture supernatant containing various metabolites and cellular components, which could inhibit TNF-α production in the LPS-activated THP-1 assay. Furthermore, this strain minimally stimulated TNF-α expression in direct contact with the THP-1 cells, suggesting that it may be less inflammatory by itself in the gut (Figs. 1 and 2). Taken together, we postulated that BM36301 is an anti-inflammatory probiotic. In order to evaluate the in vivo effects of the strain, we examined various body measures of the mice that had been fed with this strain in drinking water. During the natural aging process, the treatment with *L. reuteri* BM36301 helped the male mice maintain reduced weight gain by 36 % less than the control mice (Fig. 4). Also, this *L. reuteri* treatment resulted in males with heavier testicles and significantly higher serum testosterone levels. BM36301-fed female mice were found to have significantly lower levels of serum TNF-α than the control (Fig. 3). In addition, females consuming this strain displayed healthier skin with active folliculogenesis and faster hair growth (Fig. 5).

Identification of the anti-inflammatory metabolites from probiotic strains is being actively pursued due to their pharmaceutical effects. More recently, it was shown that histamine metabolically secreted into culture media from the human-commensal, *L. reuteri* ATCC PTA 6475, could effectively suppress a TLR pathway in vitro, which is involved in the TNF-α stimulation by LPS [23, 39]. This strain turned out to be an effective probiotic from in vivo trials with experimental models in regard to colitis, IBD, obesity, aging, and skin health [36, 38, 40, 41]. In our study, the components responsible for TNF-α suppression from the BM36301 in CM appeared to be heat-stable, non-protein molecules (Fig. 2). It is of future interest to isolate these molecules and further analyze their biochemical properties. It would also be ultimately important to identify their biological activities related with the various health benefits we have discovered in this study.

Unlike BM36301, another *L. reuteri* strain BM36304 did not produce such an anti-inflammatory CM under our experimental conditions. Instead, BM36304 cells stimulated THP-1 to produce TNF-α in vitro (Fig. 1), indicating an example of strain-specificity. However, this does not mean that BM36304 is pro-inflammatory de facto, since it is not yet clearly understood how a strain can drive immune regulations into diverse directions. For example, the *L. reuteri* strain DSM 17938, which demonstrated pro-inflammatory effects in vitro, has been previously reported for its overall anti-inflammatory effects from in vivo animal models of necrotizing enterocolitis [42]. Indeed, we have not examined the effects of BM36304 on IECs, which are actual cells in the lumen surface. Furthermore, we have not observed adverse clinical effects in mice treated with this bacteria. We may need to assess other indexes such as blood sugar or skin dermatitis scores from a BM36304-treated model for further clarification.

The probiotic benefits of BM36301 manifested differently depending on the host gender. Recent studies performed with male mice suggest that age-associated testicular atrophy is mostly due to the pro-inflammatory activation of the immune system, including T_H_17 lymphocytes [36]. Therefore, it seems likely that the anti-inflammatory network in the BM36301-fed males protected their testicle size and testosterone levels from atrophy as they aged, while the testes of untreated males displayed pro-inflammatory deterioration. Since testosterone is important for the conservation of youthful, healthy bodies during the aging process, it can be explained such that the high testosterone levels in BM36301-fed males mediated the maintenance of muscle and weight control over time. In females, feeding BM36301 lowered serum TNF-α significantly, a consequence which may be related with healthier skin [21, 38]. It is not clear why BM36301 conferred such gender-dependent benefits in these distinctive health categories, but gender is indeed known to have profound effects on skin health, immune system, and disease [43]. Moreover, it has been reported frequently that host gender is a crucial determinant of probiotic effects. For example, an application of the *L. reuteri* ATCC PTA 6475 on mice has elicited gender-dependent responses in TNF-α suppression and bone density [44]. More comprehensive studies with various other lactobacilli have also supported gender-dependent outcomes [45]. Likewise, it is possible that BM36301 affects male and female bona fide in different ways [21, 43].

It should also be noted, however, that some observations from the BM36301 treatment were barely distinguishable from those of the control. For example, the diameter of seminiferous tubules in the control male testes was not significantly different from that of the treatment group. Also, hairs of the control females were as shiny as those of the BM36301-fed females. Likewise, skin depth measurements were not very different between the two groups. Furthermore, we detected relatively high populations of active hair follicle stages (anagen + catagen) in the control. Collectively, these dimensions suggest that the control mice were not experiencing serious health deteriorations at the time of observation. It is necessary to further compare any variations among the testes of male groups by examining microscopic details, such as Leydig cells or spermatogenesis. As a comparison, Erdman and her colleagues reported that the *L. reuteri* ATCC PTA 6475 presented skin health benefits to both male and female mice, even though females showed more obvious fur luster than males. In notable contrast, they observed that the control mice displayed dull fur and skin dermatitis at the age of 40 weeks [38]. However, we did not observe such skin deterioration in our control groups. Taken together, we speculate that only highly elevated measures (such as testosterone and testes in males, and TNF-α, hair re-growth, and folliculogenesis in females) might have been scored with significant differences in our experiments. These gender-dependent effects of BM36301 requires further studies with older mice.

An increasing number of reports claim that there are inflammation-mediated connections between gut microbes and disease [12, 19–21, 46]. That TNF-α has been evidenced to be involved in numerous inflammatory diseases prompted us to choose the cytokine as a biomarker throughout the study [47]. However, the inflammatory network is very complex and many other measurements should be additionally included for a more comprehensive understanding. Therefore, comparing the levels of a larger selection of cytokines together, such as IL-10, IL-12, and TNF-α, may provide further clarity upon host inflammatory status [13]. Also, direct assessment of T_H_17 cells and T_Reg_ cells upon probiotic treatments may reveal a better inflammatory rank. Finally, utilizing knockout mice of specific cytokines such as IL-10 or IL-17, would clarify the roles of such intervening molecules in the mechanism of conveying those probiotic benefits from the gut to the immune network. Because the pro-inflammatory T_H_17 cells preferentially accumulate in the intestine, it has been postulated that the development of T_H_17 cells is regulated mainly by gut microbes [19, 20]. Therefore, anti-inflammatory probiotics may present health benefits through IL-10-dependent induction of the adaptive immune activities such as CD4^+^CD25^+^Foxp3^+^ T_Reg_ cells, which in turn operate to downregulate IL-17 production from T_H_17 cells [1, 13, 19, 21, 36, 41]. Thus it is of relative interest to study how the probiotic BM36301 can modulate these subsets of lymphocytes and cytokines in vivo.

## Conclusion

In summary, we introduce a new human-derived strain, *L. reuteri* BM36301, which shows preliminary evidence for anti-inflammatory activities both in vitro and in vivo. The in vitro screening method to select bacteria by CM in combination with live cell treatments seems to be a valid starting point for further studies toward animal applications. Current data suggest that BM36301 may fall into a category of probiotics, which confers distinct health benefits to its host in a gender-dependent manner.

## Methods

### Bacterial cultures, human cell lines, media, and chemicals

We isolated all the LAB used in this study from human feces (Table 1). The lactobacilli were cultivated in deMan, Rogosa, Sharpe (MRS; 3 M Health Care, St. Paul, MN) media and the bifidobacterium was in BL media (Acumedia, Lansing, MI). Anaerobic conditions were generated with sachets of AnaeroPack-Anaero (Mitsubishi Gas Chemical, Japan) in an air-tight jar. At 24 h of cultivation in liquid media, the *L. reuteri* BM36301 and BM36304 reached 1.3 × 10^9^ colony forming units per 1 ml (CFU/ml), *L. gasseri* BM33601 to 2 × 10^8^ CFU/ml, and *B. animalis* subsp. *lactis* BM10307 to 1 × 10^9^ CFU/ml. The physical cell count of the *L. reuteri* strains, measured on a Hemacytometer, was about 1.1 × 10^9^/ml from this routine, indicating that most of the cells were intact enough to be able to produce colonies. The *Escherichia coli* strains of DH10b and DH5Α were grown in Luria-Bertani (LB) medium. The human cell lines THP-1 (TIB-202) and HT-29 (HTB-38) were purchased from ATCC (Manassas, VA), and maintained at 37 °C under 5 % CO_2_ in RPMI-1640 medium (ATCC) and in McCoy’s 5A modified medium (Life Technologies, Carlsbad, CA), respectively. These media were supplemented with 10 % fetal bovine serum and 100 U/ml penicillin plus streptomycin (Life Technologies). Oxgall powder was purchased from Chem-Impex Int’l (Wood Dale, IL). The Gram staining kit was from Fisher Scientific (Pittsburg, PA). The LPS of *E. coli* 0127:B8 was obtained from Sigma (St. Louis, MO).

### Characterization of probiotic properties

#### Classification

The taxonomic classification was confirmed by sequencing the 16S rRNA and running the EZ-taxon [48] or BLAST program against public databases.

#### Acid and bile resistance

Resistance tests against acid or bile salt were performed by incubating exponentially growing bacterial cultures for 1 h at 37 °C in MRS media, with pH 2.5 for the acid challenge and MRS media containing 0.3 % Oxgall for the bile challenge, followed by plating after serial dilutions. Control cultures without these treatments were processed in parallel to calculate the survival rates.

#### Antimicrobial activity

Spots of 5 mm size in diameter with 1–2 × 10^5^ CFU inoculation from each strain were grown on MRS agar-plates for 1–2 days, followed by pouring LB soft agar containing *E. coli* cells. Clear inhibitory zones were measured from the edge of the LAB spots.

#### Adhesion assay

Adhesion assay was performed according to Bernet et al with modification [16]. In brief, HT-29 cells were seeded onto tissue culture coverslips (Sarstedt, Newton, NC) and incubated for 15–20 days with media changes on alternating days. Overnight-grown bacterial cultures of 1–5 × 10^8^ CFU were applied on the coverslip for 1 h at 37 °C and 5 % CO_2_. After washing them five times with phosphate buffered saline (PBS, pH 7.4), adherent bacteria were fixed with methanol and visualized by Gram staining. The bacterial cells from 20 random microscopic views were counted and the procedure was repeated more than three times. The average adherent bacterial counts per HT-29 cell were shown.

### Measurement of TNF-α from THP-1 cells treated with CM or live cells

For the preparation of the CM, LAB cells were grown in glucose-based media such as MRS or BL to the stationary phase (up to 24 h) in a standing culture at 37 °C. Cells were cleared by spinning at 4000 × *g* for 10 min. The supernatant collected was filter-sterilized (0.2 μm, Corning, NY), vacuum-dried, and resuspended in an equal volume of RPMI-1640 [23]. The THP-1 cells of 5 × 10^4^ in 1 ml of culture volume were stimulated with 150 ng/ml of *E. coli* LPS for 3.5 h at 37 °C with 5 % CO_2_. The CM was added to 5 % (v/v) prior to the LPS addition when needed. Cultures were collected and the supernatant was used to measure TNF-α quantitatively by ELISA method according to the manufacturer’s instruction (Ready-Set-Go kit, eBioscience, San Diego, CA). For physicochemical stability, the CMs were boiled for 10 min or treated with trypsin (0.25 mg/ml) for 30 min at 37 °C. For the live cell treatment, LAB cells were grown exponentially for 16 h, collected as above, washed once with PBS, and applied to 6 × 10^5^ THP-1 cells in 250-fold excess [31]. After 6 h of incubation at 37 °C with 5 % CO_2_, the cultures were collected and the supernatants were used to measure TNF-α as described above.

### Animal experiments

#### Mouse

Eighteen week old specific pathogen-free C57BL/6 mice were acquired from The Jackson Laboratory (Bar Harbor, ME) and housed in a facility accredited by the Association for Assessment and Accreditation of Laboratory Animal Care (AAALAC), International. A total of 24 female and 18 male mice were housed in socially compatible groups in individually ventilated cages. Mice were fed ad libitum Teklad Global 14 % Protein Rodent Maintenance Diet (Teklad, Madison, WI). The room in which the mice were kept was maintained under at a 12:12 light: dark cycle. The mice were acclimated to these conditions for 2 weeks prior to beginning experimental use. All studies were approved by the University of Notre Dame Institutional Animal Care and Use Committee.

#### Treatment groups

At 20 weeks of age, the mice cages were randomly placed into three treatment groups (6 males and 8 females per group) for control, BM36301, and BM36304. The mice were closely monitored to establish that they consumed normal volumes of water. They were weighed every week.

#### Probiotic preparations

Lyophilized preparations of BM36301 and BM36304 were added to water bottles containing 250 ml of reversed osmosic (RO) water. Preparations were readily reconstituted and the fresh water bottles were supplied every day to the mice cages as a source of drinking water. The control group mice received only RO water with dextrose, the cryoptotectant. The amount of bacteria to be used was adjusted for the reconstituted water supply so that each mouse can consume about 1 × 10^6^ live bacteria daily for 20 weeks. The viable bacterial counts were verified during the course at times.

#### Assessment of hair coat

To evaluate the effect of bacterial treatment on the hair coat, the hair luster and hair regrowth were assessed at times. To evaluate hair regrowth, a 2 × 2 cm area of hair was shaved from the dorsum of the mice after 18 weeks of LAB treatment. The hair luster of each mouse was measured at the 19^th^ week of LAB treatment in a dark room with no outside light contamination. The light source consisted of one 50-watt halogen bulb delivering 430 lumens. The light meter was a Sekonic Flashmate (model L-308S) with a Lumidisc sensor installed for capturing the light only directly reflected into the detector, thereby eliminating contamination from the light source. The ISO of the Flashmate was set to 100. We fabricated a base and supports to maintain consistent distance, light source angle, and detector angle.

#### Euthanasia and collection of serum

Twenty weeks after initiation of treatment (40 weeks of age), the mice were euthanized by exsanguination while under deep carbon dioxide narcosis. Blood was sampled by percutaneous cardiac puncture and the serum was separated by centrifugation. To ensure death of the animals, bilateral pneumothorax was performed.

#### Necropsy and histopathology

Following euthanasia, necropsy was performed on each mouse to identify any gross abnormalities. Samples of skin from the area that had been shaved from each mouse were placed in both 10 % neutral buffered formalin (NBF) and liquid nitrogen. Testicles were weighed in pairs from each mouse and placed in NBF. Formalin-fixed tissues were sectioned at 3–5 μm and stained with Hematoxylin and Eosin in preparation for histological examination. Skin samples were evaluated microscopically for depth of the epidermis to the panniculus carnosus. The fields chosen for measuring skin thickness were qualified by the angle of the section; that is, any section that was not an even cut through the entirety of the tissue and where the thickness of the panniculus carnosus was not similar throughout, was disqualified from inclusion. For each mouse, five measurements were taken. In addition, the number of hair follicles were counted with a minimum of five representative high-power fields per sample. Hair follicle staging was performed in a minimum of 30 longitudinally-oriented hair follicles per experimental group. Follicles were staged for H & E stained sections using the morphological criteria as previously described [49]. Testicles were examined microscopically and the diameters of ten cross-sections of seminiferous tubules were measured for each mouse. Tissue blocks were oriented to have most of the seminiferous tubules presented in cross section. Diameters of tubules were measured across the minor of their profiles (Additional file 1). All the pictures were taken with a SPOT RT digital camera (software v3.5) mounted on the Zeiss Axioplan microscope.

#### Measurement of serum cytokines, testosterone, and insulin

The TNF-α and IL-10 levels from the mouse serum were measured with the mouse TNF-α high sensitivity ELISA kit and the mouse IL-10 platinum ELISA kit, respectively (eBioscience). Insulin was quantified with the ultra-sensitive mouse insulin ELISA kit (Crystal Chem Inc., Downers Grove, IL). Serum testosterone was measured with the ELISA kit from Enzo Life Science (Farmingdale, NY).

### Statistical analysis

Pairwise comparisons from multiple mouse data were performed by the Tukey test of one-way ANOVA method using the GraphPad Prism 6 program (GraphPad Software, San Diego, CA). Two data set comparison was confirmed by student t-test using the Excel program (Microsoft, Richmond, WA). Any *p* values smaller than 0.05 were considered statistically meaningful.

### Ethics approval and consent to participate

Mice were housed in a facility accredited by the Association for Assessment and Accreditation of Laboratory Animal Care International, and all related studies were approved by the University of Notre Dame Institutional Animal Care and Use Committee. The donor of the fecal samples which were analyzed in the study has provided consent to use the contents of the samples and their isolates for the purposes of this paper.

## Additional file

Additional file 1: Supplementary information. (DOCX 3849 kb)

## Abbreviations

BM: Benebios Microorganism
CM: conditioned medium
ELISA: enzyme-linked immunosorbent assay
HF: hair follicle
IBD: inflammatory bowel disease
IEC: intestinal epithelial cell
IL: interleukin
LAB: lactic acid bacteria
LPS: lipopolysaccharide
TLR: toll-like receptor
TNF: tumor necrosis factor
