# Supplementary material for: Characterization of the anti-inflammatory Lactobacillus reuteri BM36301 and its probiotic benefits on aged mice
Source: BMC Microbiol. 2016 Apr 19;16:69. doi: 10.1186/s12866-016-0686-7 (PMC4837529; doi:10.1186/s12866-016-0686-7)
Supplement: Additional file 1: — Supplementary information. (DOCX 3849 kb) [file 12866_2016_686_MOESM1_ESM.docx]

**Supplementary Information**

**S1. Change of THP-1 dead cells during treatment with LAB.**

| LAB treated | Dead Cells (%) in total THP-1 after incubation | |
| --- | --- | --- |
|  | 0 hr | 6 hr |
| No addition | 2.45 ± 0.79 | 2.17 ± 0.73 |
| BM36301 | 1.89 ± 0.83 | 9.73 ± 2.75 |
| BM36304 | 2.15 ± 0.62 | 25.42 ± 3.07 |
| BM33601 | 1.81 ± 0.42 | 23.52 ± 5.75 |
| BM10307 | 1.93 ± 0.81 | 38.24 ± 5.84 |

The control (no treatment) maintained the basal dead cell population (about 2%) throughout the co-incubation. We confirmed similar dead cell populations at the beginning of all the treated co-incubations (0 hr). Upon a 6-hr treatment, BM36301-treated THP-1 cells showed an increase of dead cells to 9.73%. Meanwhile, THP-1 cells treated with BM36304 or BM33601 showed more increased dead cell populations (to about 24%). Finally, the BM10307 treatment resulted in a notably marked increase of THP-1 dead cells (to 38%). The dead cell populations are largely correlated with the amount of TNF-α produced; that is, BM36301 induced the smallest amount of TNF-α, BM36304 and BM33601 induced a higher amount of TNF-α, and BM10307 induced the highest amount of TNF-α (Figure 1B). Therefore, it is not likely that BM36301 induced lower TNF-α because of the loss of THP-1 viability. It is not clear how the treatment of LAB caused THP-1 death. One possibility is that the TNF-α produced by the THP-1 cells as a result of treatment caused their own death. Another possibility is that the bacterial cells included directly kill the THP-1 cells; however, we included antibiotics in the culture media, which should have kept the bacterial metabolism minimally low.

*Method* – About 4×10^5^ THP-1 cells in 1 ml of culture media were treated with live bacterial cells of 250-fold higher count (1×10^8^ CFU) at 0 hr and further incubated for 6 hr. At each time point, aliquots of the co-incubations were taken and the THP-1 cells were counted on the Hemacytometer after staining with Trypan-Blue (0.2% final for 10 min, Life Technologies, Carlsbad, CA). Cells stained blue were considered dead and included in the dead cell population as a percentage of the total cell coints. From two independent experiments, four aliquots were counted to have average ± standard deviation.

**S2. Comparison of skin cross sections from control mice and BM36301-treated mice.**


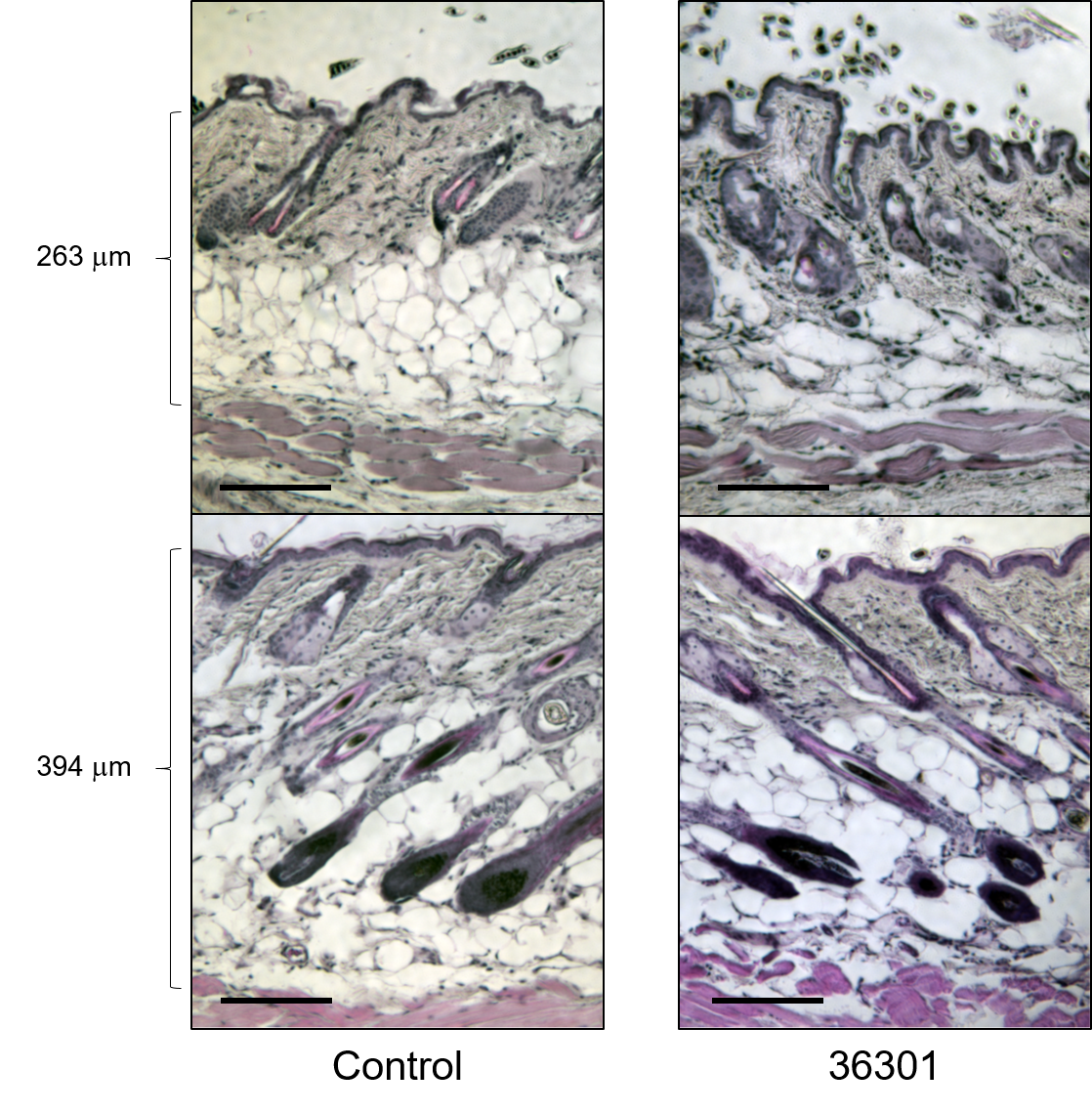


Skin cross sections prepared from control mice (left) and BM36301-treated mice (right) were stained with Hematoxylin and Eosin, and photos were taken at 100× resolution. The bar indicates 100 μm in length. The skin depth was measured from images with intact skin structure and similar thickness of the panniculus carnosus. We aligned two representative images with similar HF stages from each mouse. The measurements are indicated on the left.

**S3. Measurement of the diameter of seminiferous tubules.**


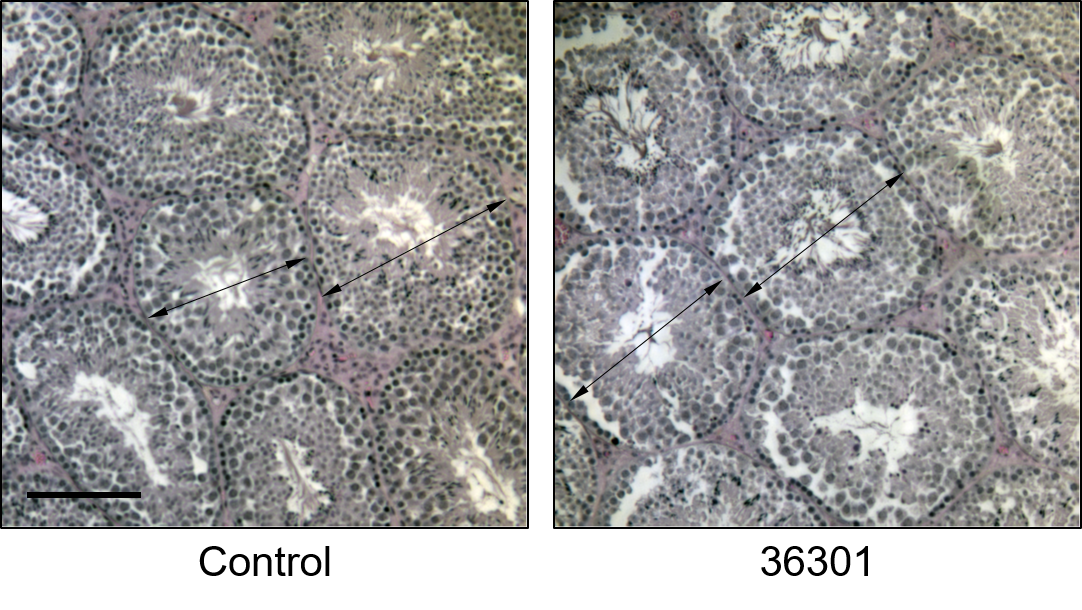


Seminiferous tubule (ST) cross-sections from the control mice (left) and BM36301-treated mice (right) were stained with Hematoxylin and Eosin. Photos were taken at 100× resolution from tissue blocks oriented to have the ST presented in the cross section. For the diameter of ST, minor axes of ten profiles randomly selected from each mouse were measured according to Moffit et al [1]. Six male mice were used per group. Representative images from each group were shown with arrows measuring the minor axes of the profile. The bar indicates 100 μm in length.

1. Moffit JS, Bryant BH, Hall SJ, Boekelheide K: **Dose-dependent effects of sertoli cell toxicants 2,5-hexanedione, carbendazim, and mono-(2-ethylhexyl) phthalate in adult rat testis**. *Toxicol Pathol* 2007, **35**(5):719-727.
